# Editorial: Food, nature & wellness: dueling epistemologies

**DOI:** 10.3389/fpubh.2023.1264967

**Published:** 2023-10-24

**Authors:** Debbie Humphries, Alder Keleman Saxena, Padma Venkatasubramanian

**Affiliations:** ^1^School of Public Health, Yale University, New Haven, CT, United States; ^2^Department of Anthropology and Program in Sustainable Communities, Northern Arizona University, Flagstaff, AZ, United States; ^3^School of Health Sciences and Technology, University of Petroleum and Energy Studies, Dehradun, India

**Keywords:** food, nature, wellness, food system, ecosystem health

## Introduction

Who is responsible for the health of the food system? In today's globally interconnected world, the wellbeing of land, labor, water, and air are generally invisible to most consumers. Industrialized food systems negatively impact natural resources and human health, causing diet-related diseases, foodborne diseases, and long-term, cumulative effects of pesticide residues.

Producing and distributing food that sustains ecosystem fertility and nourishes consumer health requires attention to a breadth of issues, covering many disciplines and communities of practice. While interdisciplinary research in this area is increasing, addressing the full complexity of food system wellbeing requires prioritizing vital and robust life at every stage, and stressing the interconnectedness between the health of humans and that of the planet.

In this Research Topic, we bring together articles addressing different dimensions of food, nature, and wellness, including knowledge frameworks (de Garine-Wichatitsky et al.; Timotijevic et al.; Spring et al.; Ramenzoni), limits of current methodologies in assessing these domains (Hemsworth et al.; Saxena et al.), policy approaches to strengthening human and ecosystem wellness in the food system (Canfield et al.; Valencia et al.; Roothaert et al.; Queenan et al.), and the importance of connection with the natural world for human mental health (Lengieza and Swim; Thiermann et al.; Venkatasubramanian). Together these articles emphasize the importance of mindfulness and interdisciplinary research to advance science and knowledge at the food systems-ecology-human wellbeing nexus.

## Mindfulness practices for wellness and sustainability

Connectedness with nature (CWN) is an important construct for appreciating the intricate and complex interdependence of people and the planet. CWN is fundamental to discussions on Climate Change/ Planetary Health/ Sustainable Development Goals. Three articles in this Research Topic discuss practices that promote mindfulness and pro-environmental activities (Lengieza and Swim; Thiermann et al.; Venkatasubramanian). Lengieza and Swim review indicates that self-awareness and mindfulness are associated with CWN, which is influenced by age, openness to experience, worldviews toward nature, and self-transcendence. Through a study of 300 individuals, Thiermann et al. have shown that advanced meditators reported significantly more pro-environmental activities and the lowest greenhouse gas emissions related to their non-animal protein diet when compared to non-meditators. The authors suggest that CWN is an innate human quality that is affected by modern-day pressures (Thiermann et al.). Venkatasubramanian delineates a novel pedagogical approach in a University Wellness Program (UWP) that nudges students' behavior toward wellness and campus sustainability. Experiential learning of academic modules and evaluation systems are leveraged for students to design and implement projects that are relevant to campus. These authors suggest CWN is an innate human quality that strengthens individual interest and attention to ecosystem and food system health.

## Potential role of different knowledge frameworks

Four articles address frameworks for thinking about how and what we know, including conceptualizing responsibility in the food system (Timotijevic et al.), integrating human and ecosystem considerations in addressing socio-ecological system health and resilience (de Garine-Wichatitsky et al.), listening more deeply to cultural reasons for food avoidance to better inform nutritional interventions (Ramenzoni), and using the insights from different ways of knowing to strengthen community food security (Spring et al.). Timotijevic et al. utilize in-depth interviews with individuals working in research, civil society, policy, and industry on cutting-edge approaches in the food system that may impact health and identify four overlapping conceptualizations of responsibility: accountability, impact, reflexivity, and responsiveness. de Garine-Wichatitsky et al. develope a participatory five-step approach to framing and taking action to strengthen socio-ecological system resilience that includes human health, animal health, plant health, and environmental health, and then pilot the framework and approach with an agro-ecological systems project in Vietnam (de Garine-Wichatitsky et al.). Ramenzoni utilizes a mixed-methods approach to study food avoidances among the Coastal Endenese ethnic group, observing that certain types of foods are avoided out of concern for upsetting cosmological relationships, and urging a more community-based and collaborative approach to nutrition interventions. In Spring et al. community-partnered research on food security in the Tsá Tué Biosphere Reserve (Northwest Territories, Canada), the authors examine the interaction between traditional food systems and climate change using a community capitals framework, underscoring the potential of traditional foodways to buffer risks. Taken together these articles highlight the value of conceptual frameworks for addressing complex issues with greater cultural awareness and sensitivity and emphasize the need to include non-Western frameworks.

## Methodologies for assessing components of the agricultural system

Two articles highlight methodological challenges in studying food, nature, and wellness, whether in assessing population-level perspectives on animal welfare (Hemsworth et al.) or in exploring the role of traditional foods in food security and nutritional status (Saxena et al.). Hemsworth et al. explore current methodologies for generating representative perspectives of populations by comparing perspectives on animal welfare from a computer-assisted telephone interview of >500 respondents and a probability internet panel of >500 respondents, identifying important differences in respondent attitudes and experiences across the two methods. Saxena et al. explore methodological approaches for assessing connections between agrobiodiversity and food security, demonstrating that standard dietary intake methodologies may underestimate the impacts and importance of traditional foods. Both studies draw attention to the importance of careful conceptualization of conclusions to ensure they are fully supported by the data and the need for innovative methods and analytical approaches to better capture complex systems.

## Importance of policy in enhancing food system sustainability

Another key theme in these articles is the importance of public policy (and public funding) for food system sustainability. Based on data from Brazil, Valencia et al. argue that integrating sustainability into public food procurement systems can help to support other sustainable development goals, including poverty reduction, zero hunger, and gender equity. Examining the potential for the uptake of a model similar to Brazil's, Roothaert et al. explore the feasibility of expanding Home-Grown School Feeding programs in Tanzania and conclude such expansion might address some systemic issues, but will not fully substitute for a larger policy framework and resource investment in school meals. Systemic challenges are also the subject of Queenan et al. research, which identifies issues such as an unlevel playing field for importers and local producers, and a small number of large-scale producers with a large market share, that inhibit the potential of the commercial broiler system in South Africa to contribute to the sustainable development goals. Turning to the international scene, Canfield et al. document how the organization of the 2021 UN Food Systems Summit sidestepped existing forums for accountable multilateral food system governance, demonstrating the corporate capture of the international public system's governance of agriculture. Each of these articles highlights the potential of particular policies to enhance wellness.

## Conclusions: avoiding pitfalls in new ways forward

Both frameworks and methodologies offer the potential of clear research approaches that are consistent across research studies allowing for better comparison of results. They can also be applied to different contexts and disciplines which may highlight strengths and weaknesses. However, adopting specific knowledge frameworks /methodologies could lead to an overemphasis on the frameworks/methodologies rather than on empirical observations. This can lead researchers to disregard findings that do not fit their pre-determined categories of analysis. Recommendations include being explicit about frameworks and methods, with clear definitions of constructs, moving beyond past biases by incorporating and adapting multiple frameworks and multidisciplinary methods wherever possible, and using mixed methods and participatory approaches with due consideration of local knowledge, that are better able to honor and characterize complex local systems. Key limitations include the absence of mechanisms to encourage the adoption of specific frameworks and the emphasis on innovation in academic institutions that rewards individual researchers for developing their own frameworks, rather than using and testing the frameworks of others.

The articles on policy and practices illuminate both the opportunities and limitations. There are opportunities for increased use of intersectoral and intersectional policy frameworks and processes. Mindfulness and CWN may provide ways to get beyond business as usual, as a tool for transformative individual, research, and systems change. In addition, similar to the challenges with frameworks and methodologies, there are only limited levers to encourage and reward multidimensional approaches. Taken together, these articles break ground toward reconceptualizing food system health, and locating responsibility for food system outcomes—but they also highlight that much remains to be done to achieve holistic food system health for people and planet. To promote such multidimensional approaches will require flexibility and re-invention not only of research questions and methods, but also of the institutions and funding structures that bring such research into being.

## Author contributions

DH: Conceptualization, Writing—original draft, Writing—review and editing. AS: Conceptualization, Writing—original draft, Writing—review and editing. PV: Conceptualization, Writing—original draft, Writing—review and editing.

